# Oral Methylthioadenosine Administration Attenuates Fibrosis and Chronic Liver Disease Progression in *Mdr2−/−* Mice

**DOI:** 10.1371/journal.pone.0015690

**Published:** 2010-12-29

**Authors:** M. Ujue Latasa, Carmen Gil-Puig, Maite G. Fernández-Barrena, Carlos M. Rodríguez-Ortigosa, Jesús M. Banales, Raquel Urtasun, Saioa Goñi, Miriam Méndez, Sara Arcelus, Nerea Juanarena, Juan A. Recio, Sophie Lotersztajn, Jesús Prieto, Carmen Berasain, Fernando J. Corrales, Jon Lecanda, Matías A. Ávila

**Affiliations:** 1 Division of Hepatology and Gene Therapy, CIMA, University of Navarra, Pamplona, Spain; 2 Digna Biotech, Madrid, Spain; 3 CIBERehd, University Clinic, University of Navarra, Pamplona, Spain; 4 Vall d'Hebron Research Institute, Institute of Oncology and Hospital, Barcelona, Spain; 5 Inserm, U955, Créteil, France; 6 Université Paris-Est, Faculté de Médecine, UMR-S955, Créteil, France; University of Tor Vergata, Italy

## Abstract

**Background:**

Inflammation and fibrogenesis are directly related to chronic liver disease progression, including hepatocellular carcinoma (HCC) development. Currently there are few therapeutic options available to inhibit liver fibrosis. We have evaluated the hepatoprotective and anti-fibrotic potential of orally-administered 5′-methylthioadenosine (MTA) in *Mdr2^−/−^* mice, a clinically relevant model of sclerosing cholangitis and spontaneous biliary fibrosis, followed at later stages by HCC development.

**Methodology:**

MTA was administered daily by gavage to wild type and *Mdr2^−/−^* mice for three weeks. MTA anti-inflammatory and anti-fibrotic effects and potential mechanisms of action were examined in the liver of *Mdr2^−/−^* mice with ongoing fibrogenesis and in cultured liver fibrogenic cells (myofibroblasts).

**Principal Findings:**

MTA treatment reduced hepatomegaly and liver injury. α-Smooth muscle actin immunoreactivity and collagen deposition were also significantly decreased. Inflammatory infiltrate, the expression of the cytokines IL6 and Mcp-1, pro-fibrogenic factors like TGFβ2 and tenascin-C, as well as pro-fibrogenic intracellular signalling pathways were reduced by MTA *in vivo*. MTA inhibited the activation and proliferation of isolated myofibroblasts and down-regulated cyclin D1 gene expression at the transcriptional level. The expression of JunD, a key transcription factor in liver fibrogenesis, was also reduced by MTA in activated myofibroblasts.

**Conclusions/Significance:**

Oral MTA administration was well tolerated and proved its efficacy in reducing liver inflammation and fibrosis. MTA may have multiple molecular and cellular targets. These include the inhibition of inflammatory and pro-fibrogenic cytokines, as well as the attenuation of myofibroblast activation and proliferation. Downregulation of JunD and cyclin D1 expression in myofibroblasts may be important regarding the mechanism of action of MTA. This compound could be a good candidate to be tested for the treatment of (biliary) liver fibrosis.

## Introduction

Liver diseases are currently the fifth cause of mortality in the Western world, and as opposed to other major causes of mortality their incidence is increasing [1], [2]. The principal causes of the most common liver ailments are well known, and include chronic alcohol consumption, infection by hepatitis viruses, genetic conditions such as hemochromatosis or α1-antitrypsin deficiency, autoimmune hepatitis and metabolic disorders [1], [2]. Regardless of the etiology, chronic tissue injury and inflammation are increasingly recognized as driver mechanisms in liver disease progression from fibrosis, characterized by the excessive accumulation of extracellular matrix (ECM), to hepatocellular carcinoma (HCC) [1]–[3]. The molecular links in the so-called inflammation-fibrosis-cancer axis in the liver are currently being elucidated in experimental models of acute and chronic injury. These links include a variety of intracellular pathways triggered by extracellular mediators like the cytokines interleukin 1 (IL1), IL6 and tumour necrosis factor alpha (TNFα), platelet-derived growth factors and transforming growth factor beta (TGFβ), among others [3]–[5]. These growth factors and cytokines stimulate ECM synthesis by the activation of hepatic stellate cells and myofibroblasts in the hepatic parenchyma [3]–[5]. Targeted interference with these and other mediators has clearly shown a beneficial effect on the course of the experimental disease, lending support to their pathological role [6], [7]. However, in spite of these remarkable advances the availability of safe and efficacious therapies to halt the progression of fibrosis and liver disease in humans is still limited [8], [9]. The use of experimental models of chronic liver injury resembling the human pathology is important to evaluate candidate drugs with chances of succeeding in the clinical setting. One such experimental model are the *Mdr2/Abcb4*-deficient mice (*Mdr2^−/−^*), which lack the canalicular phosphatidylcholine flippase [10]. The absence of phosphatidylcholine from bile occurring in these mice leads to bile regurgitation into the portal tracts, causing periportal inflammation and injury early in life (2–3 weeks), periportal fibrosis (4 weeks), and the appearance of preneoplastic lesions (at 4–6 months) [10]–[12]. These pathogenic characteristics resemble what occurs in human primary sclerosing cholangitis and biliary fibrosis, making these mice an excellent model to study disease mechanisms, and a test ground for hepatoprotective and antifibrotic therapies [10]–[13]. In analogy to the situation found in humans, protracted injury and inflammation lead to HCC development in virtually 100% of *Mdr2^−/−^* mice by 16 months [14].

We and others have previously demonstrated the *in vivo* anti-inflammatory effects of 5′-methylthioadenosine (MTA), a sulphur-containing adenine nucleoside produced from S-adenosylmethionine (SAMe) during polyamine biosynthesis [15]. Parenteral MTA administration reduces the production of inflammatory mediators triggered by bacterial lipopolysaccharide or pro-inflammatory peptides in mice [16]–[18], and protects from liver injury in rats treated with CCl_4_ or with the carcinogen diethylnitrosamine [19], [20]. MTA is therefore a compound with an attractive pharmacological profile for the treatment of chronic liver disease. Using the *Mdr2^−/−^* mice as a model, here we show for the first time that orally administered MTA attenuates liver injury and inflammation, and inhibits the progression of hepatic fibrosis. The mechanism of action of MTA is likely to be multifaceted. MTA reduced the production of inflammatory and pro-fibrogenic mediators and inhibited the proliferation and activation of fibrogenic cells. These observations gathered in the *Mdr2^−/−^* mouse model indicate that MTA could be useful for the treatment of biliary fibrosis and conditions such as primary sclerosing cholangitis, a severe disease frequently associated with hepatobiliary cancer and with limited therapeutic options.

## Methods

### Ethics statement

Animals received humane care and study protocols complied with our institution's guidelines and the recommendations of the European Accreditation of Laboratory Animal Care. The protocol was approved by the Committee on the Ethics of Animal Experiments of the University of Navarra (protocol number: 025-10).

### Animal studies

Male *Mdr2^−/−^* and *Mdr2^+/+^* mice (The Jackson Laboratory, Bar Harbor, ME) were fed standard laboratory diet. Two groups of mice per genotype were established (2 months old, n = 8 per group). One group received a daily oral dose of 30 mg/Kg body weight of MTA dissolved in 0.2% dimethylsulfoxide by gavage for three weeks, the control group received the same volume of vehicle (0.2% dimethylsulfoxide). MTA was from Enantia S.L. (Barcelona, Spain). This dose of MTA has been used previously, but was administered intraperitoneally [16], [17]. Liver enzymes and bilirrubin levels were analyzed in serum (Cobas® analyzer, Roche). Livers were excised, weighted, and were either snap frozen or fixed in formalin and paraffin-embedded.

### Metabolite measurement in liver tissues

Glutathione (GSH) was measured using the Gluthatione Assay Kit (Sigma, St. Louis, USA). Tissue samples were processed as recommended by the manufacturer. SAMe was measured as previously described [21].

### Tissue staining and immunohistochemistry

Tissue sections were stained with Picro-Sirius Red to visualize collagen deposition as reported [22]. For morphometric analysis images were captured at 100X magnification (Nikon Eclipse 1000), and percentage of collagen cover per field was estimated with an Arkon software (Arkon Resources Inc. Arcadia, CA) as described [22]. Values are the means of five fields taken from different tissue sections per mouse. Immunodetection of α-smooth muscle actin (αSMA) on liver sections was carried out as described using a monoclonal anti-αSMA antibody (#A2547) from Sigma (St. Louis, MO) [22]. Tenascin-C and Ki-67 immunostainings were performed using rabbit polyclonal antibodies from Millipore (#AB1951) (Millipore Iberica, Madrid, Spain) and Abcam (#ab833) (Cambridge, UK) respectively. Immunostaining for CD45 was performed with a rat-anti-mouse CD45 antibody (clone 30-F11, #103101) from BioLegend (San Diego, CA).

### Cell culture and treatments

Liver myofibroblasts were isolated from wild type and *Mdr2−/−* mice by collagenase perfusion and density-gradient purification in Nycodenz, and cultured in Dulbecco's Modified Eagle's medium (DMEM) containing 10% fetal calf serum (FCS) as described [23]. For [^3^H]thymidine incorporation myofibroblasts were incubated under the specified conditions for 24 h with 2 µCi/ml [^3^H]thymidine. Cells were lysed and radioactivity measured as described [22]. Human recombinant platelet derived growth factor BB (PDGF) was from Calbiochem (#521225) (Merck KGaA, Darmstadt, Germany). MTA showed no cytotoxic effects, as determined by measuring lactate-dehydrogenase in culture media using the Cytotox assay (Promega, Madison, WI). Apoptosis was measured in cultured myofibroblasts using the Cell Death Detection Assay from Roche (Barcelona, Spain) as previously described [22].

The murine monocyte/macrophage RAW 264.7 cells (American Type Culture Collection, ATCC) were cultured in DMEM supplemented with 10% FCS. Where indicated cells were treated with *Salmonella typhymurium* lipopolysaccharide (LPS) (50 mg/mL) from Sigma (#L6511, Lot#3944110). The adenosine receptor agonist 5′-(N-Ethylcarboxamido) adenosine (NECA) and the adenosine A2B receptor antagonist MRS 1754 were from Sigma, the adenosine A2A receptor antagonist ZM 241385 was purchased from Tocris Bioscience (Bristol, UK). TNFα concentration in conditioned media was measured using an enzyme-linked immunosorbent assay (ELISA) kit from BD Biosciences (San Diego, CA).

### RNA isolation and quantitative real-time RT-PCR

Total RNA was extracted using the TRI Reagent (Sigma). Real time PCR was performed using an iCycler (BioRad, Hercules, CA) and the iQ SYBR Green Supermix (BioRad) as reported [22]. Gene expression was determined using the ΔΔCT calculation as described [21], [22]. We designed all primers to distinguish between genomic and cDNA amplification and sequenced all PCR products to confirm the specificity. Primers for α1(I)-procollagen, matrix metalloprotease-13 (MMP13) and TIMP1 determination were described previously [22]. αSMA, TGFβ1 and TGFβ2 primers were as reported [11], TNFα, IL6 and inducible NO synthase (iNOS) primers were as described [17], tenascin-C primers were as reported [24]. Monocyte chemotactic protein-1 (Mcp-1) primers were: sense 5′-CCACTCACCTGCTGCTACTC-3′, antisense 5′-TTCACATTCAAAGGTGCTGAAG-3′. JunD primers were: sense 5′-CGCCCATCGACATGGACAC-3′, antisense 5′-GTTGACGTGGCTGAGGACTT-3′. Adenosine A2B receptor primers were: sense 5′-TGGCGCTGGAGCTGGTTA-3′, antisense 5′-GCAAAGGGGATGGCGAAG-3′.

### Western blot analysis

Tissues and cells were homogenized and analyzed by Western blotting as described [22]. The antibodies used were: anti-cyclin-D1 (#sc-450), anti-c-Jun N-terminal kinase (JNK) (#sc-571), anti-phospho-extracellular signal-regulated kinase (Tyr^204^) (pErk1/2) (#sc7383), anti-Smad1/5/8 (#sc-6031-R), anti-JunD (#sc-74) from Santa Cruz Biotechnology (Santa Cruz, CA); anti-phospho-JNK(Thr^183^/Tyr^185^) (#9251), anti-c-Jun (#9165), anti-phospho-c-Jun (Ser^63^) (#9261), and anti-phospho-S6 ribosomal protein (Ser^235^/Ser^236^)(#4857) were from Cell Signaling Technology (Danvers, MA); anti-phospho-Smad2 (Ser^465/467^) (#AB3849), anti-Smad2/3 (#05-914), anti-phospho-Smad1/5/8(Ser^463/465^) (#AB3848), anti-Erk1/2 (#06-182) were from Millipore (Billerica, MA). GAPDH antibodies (#MCA4739) were from AbD-Serotec (Dusseldorf, Germany).

### Chromatin immunoprecipitation (ChIP)

ChIP assay was performed in mouse myofibroblasts essentially as described [18], using anti-JunD (#sc-74x) (Santa Cruz Biotechnology) or anti-tri-methyl-H3K4 (#ab8580) (Abcam) antibodies, or a control rabbit IgG (Santa Cruz Biotechnology). The promoter region −905 to −725 of the murine cyclin D1 gene, which includes a growth factor-responsive binding site for activator protein 1 (AP1) factors [25], was analyzed by RT-PCR with the following primers: 5′-AACGAAGCCAATCAAGAAGC-3′, 5′-CAGTATCCCCCTCCTCCACT-3′. Values were normalized to average values of inputs.

### Statistical analysis

Data are means ±SEM. Analyses were performed using GraphPad Prism version 5.00 (GraphPad Software, San Diego, USA). Data were compared among groups using the Student *t* test. A *P* value of <0.05 was considered significant.

## Results

### MTA treatment reduces liver injury in *Mdr2^−/−^* mice

**Figure 1:**
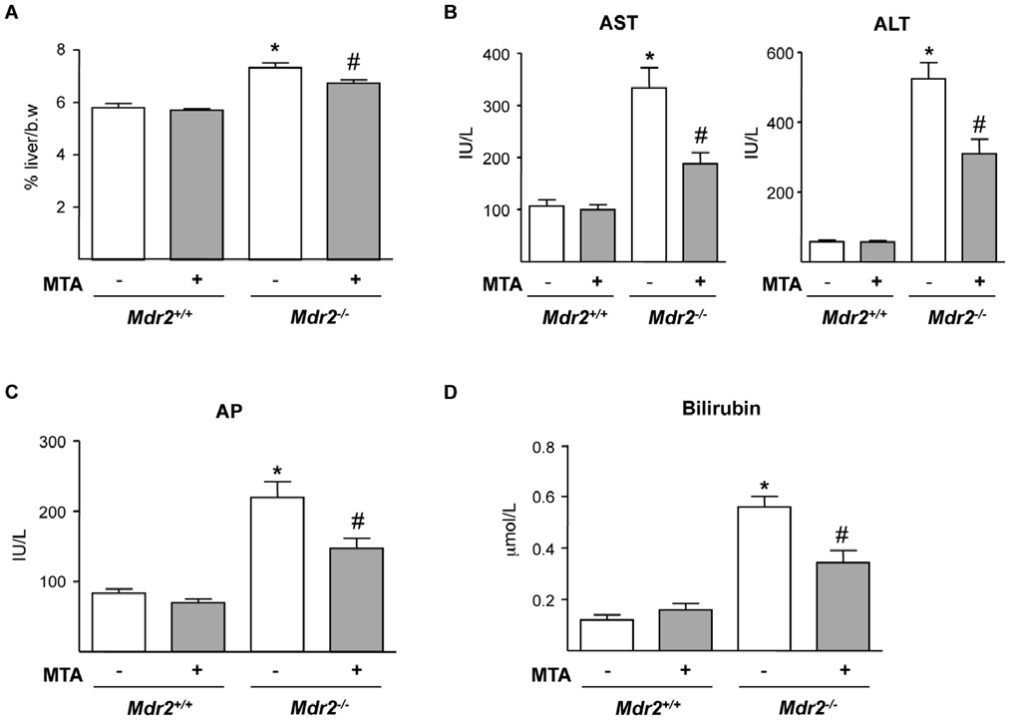
Oral administration of MTA to *Mdr2^−/−^* mice reduces hepatomegaly and serum parameters of liver injury. *Mdr2^+/+^* and *Mdr2^−/−^* mice were treated with MTA during three weeks. Increased liver-to-body weight ratio (%) in *Mdr2^−/−^* mice was reduced by MTA (A). Serum transaminases (B), alkaline phosphatase (C) and bilirubin (D), levels that are increased in *Mdr2^−/−^* mice were attenuated by MTA. *P<0.05 *vs* untreated *Mdr2^+/+^* mice, #*P*<0.05 *vs* untreated *Mdr2^−/−^* mice.

By three months of age *Mdr2^−/−^* mice typically display hepatomegaly and elevated serum levels of hepatic enzymes [10], [11], [14]. MTA administration was well tolerated, and treated animals gained similar weight as controls. MTA reduced the liver to body weight ratio (Fig. 1A), and improved aspartate aminotransferase (AST) and alanino aminotransferase (ALT) serum levels (Fig. 1B). Circulating levels of alkaline phosphatase (AP) and bilirubin were also lowered by MTA (Fig. 1C and 1D).

### MTA treatment attenuates liver fibrosis in *Mdr2^−/−^* mice

**Figure 2:**
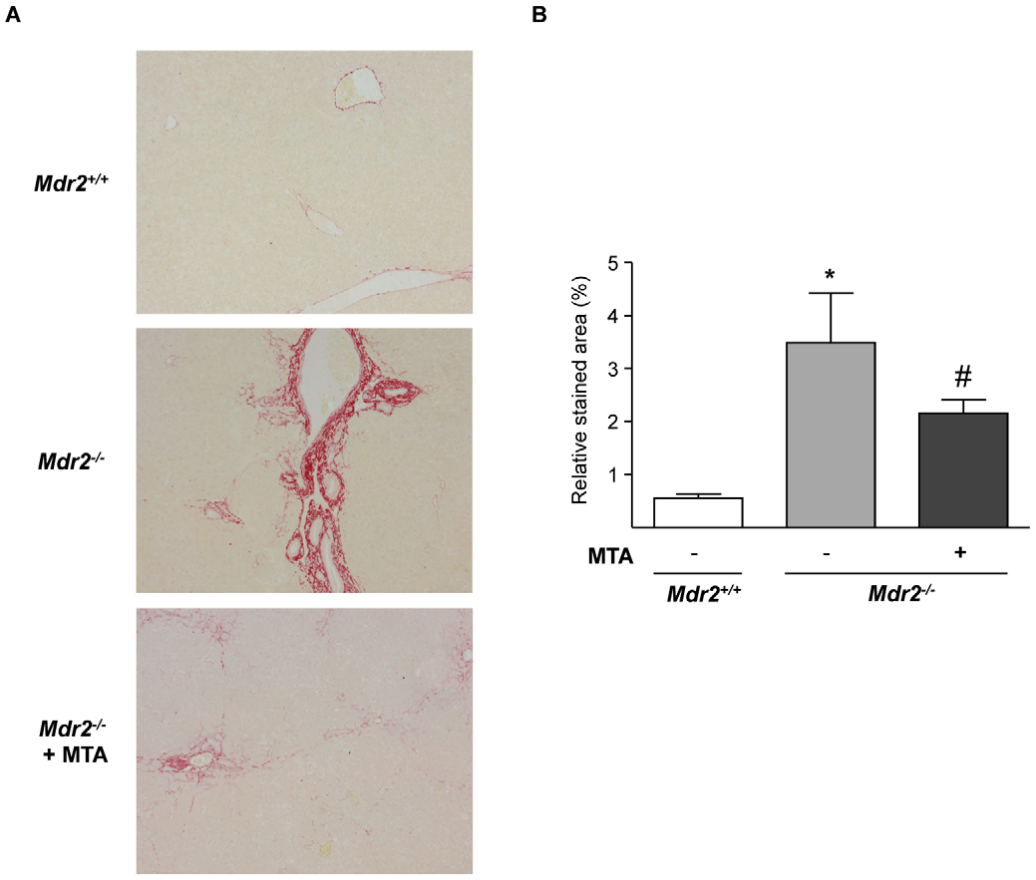
MTA administration reduces the spontaneous fibrosis that develops in *Mdr2^−/−^* mice. Representative Sirius Red-stained liver sections from *Mdr2^+/+^*, control *Mdr2^−/−^* and MTA-treated *Mdr2^−/−^* mice (*Mdr2^−/−^* + MTA) (A). Fibrosis was quantified as function of mean percentage of stained area (B). *P<0.05 *vs Mdr2^+/+^* mice, #*P*<0.05 *vs* untreated *Mdr2^−/−^* mice.

**Figure 3:**
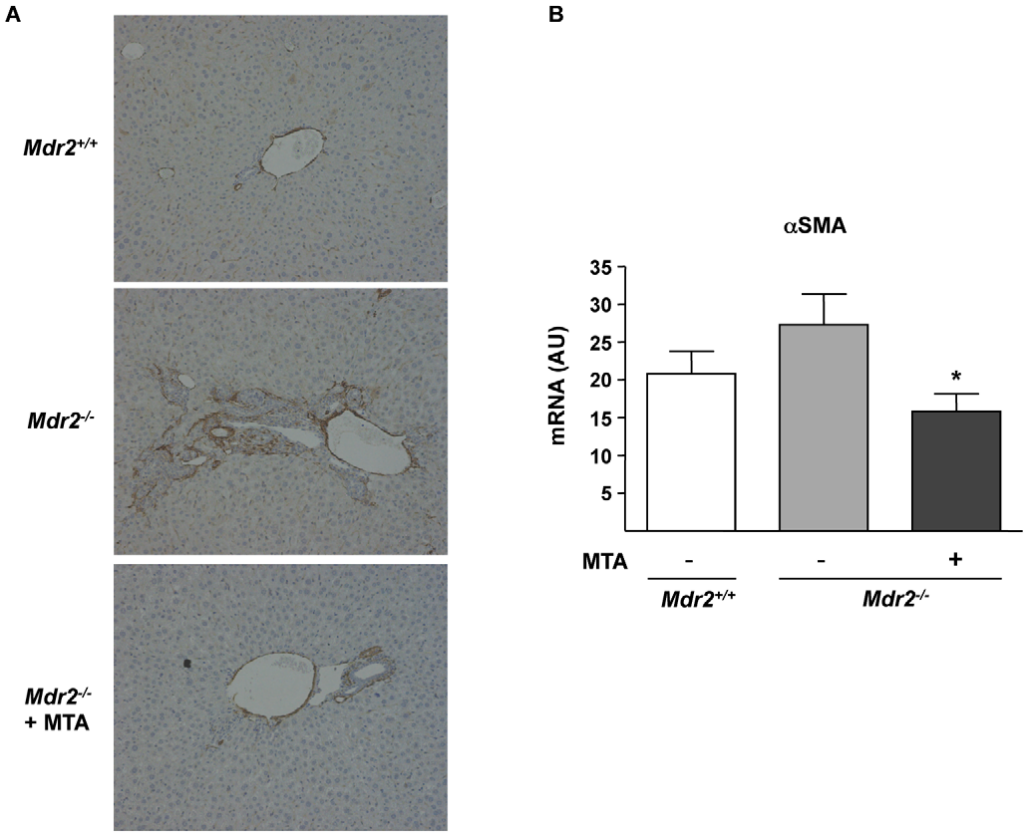
MTA administration reduces αSMA expression in *Mdr2^−/−^* mice. Immunohistochemical staining of αSMA in representative liver sections from wild type, control *Mdr2^−/−^* mice, and MTA-treated *Mdr2^−/−^* mice (*Mdr2^−/−^* + MTA). The increased number of positive periductal myofibroblasts was reduced upon MTA administration (A). αSMA mRNA levels in *Mdr2^+/+^*, control *Mdr2^−/−^*, and MTA-treated *Mdr2^−/−^* mice (B). AU: arbitrary units. *P<0.05 *vs* untreated *Mdr2^−/−^* mice.

**Figure 4:**
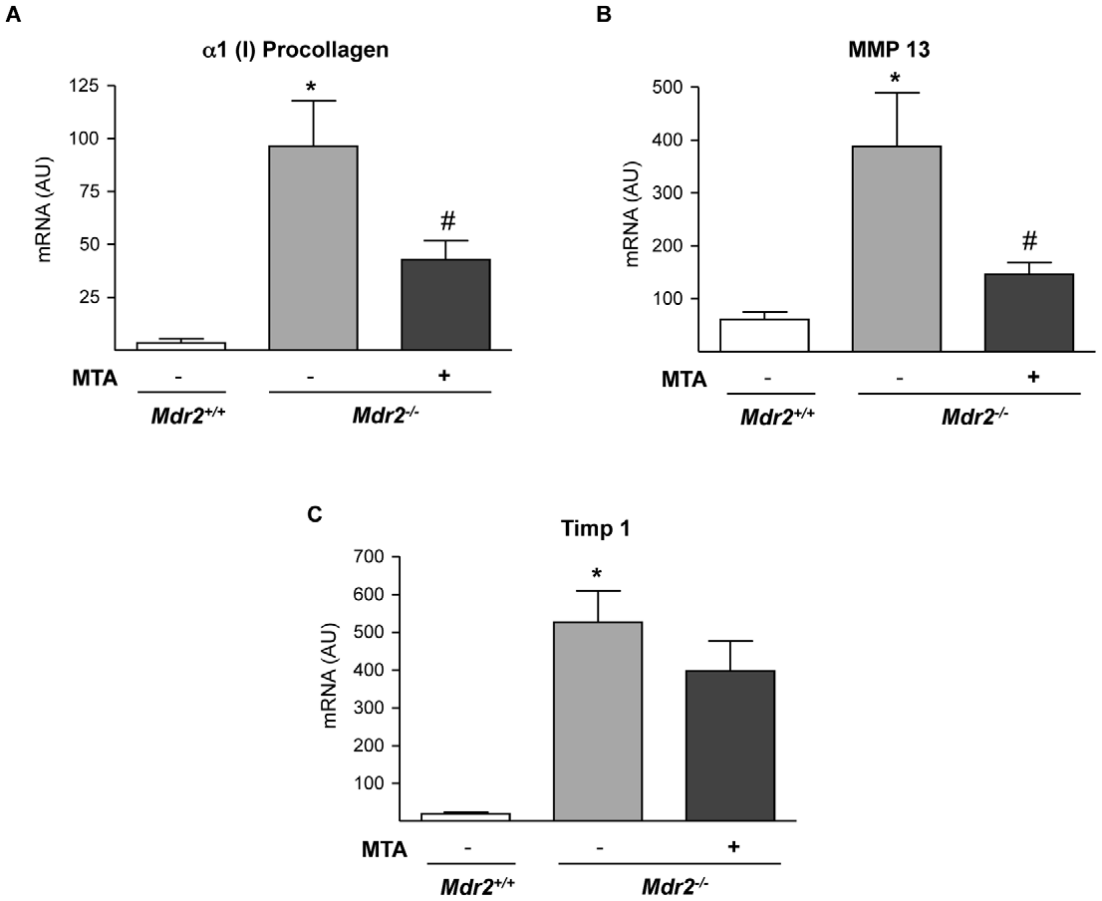
Fibrosis-related gene expression in *Mdr2^−/−^* mouse liver is attenuated by MTA treatment. mRNA levels of α1(I) procollagen (A), MMP13 (B) and TIMP1 (C), were measured in the liver of *Mdr2^+/+^*, control *Mdr2^−/−^*, and MTA-treated *Mdr2^−/−^* mice. AU: arbitrary units. *P<0.05 *vs Mdr2^+/+^* mice, #*P*<0.05 *vs* untreated *Mdr2^−/−^* mice.

MTA significantly reduced the periductal fibrosis that spontaneously develops in these mice [11], as determined by Sirius Red staining of crosslinked collagen in liver sections and morphometric quantitation of stained areas (Fig. 2A and B). MTA administration to *Mdr2* ^+/+^ mice had no effect on collagen levels (not shown). As previously described [11], we found an increase in periductal αSMA-positive cells and αSMA mRNA levels, which were also reduced by MTA treatment (Fig. 3A and B). In accordance with the histology, the expression of α1(I)procollagen mRNA was reduced in MTA treated mice (Fig. 4A). As reported [11], the expression of the interstitial collagenase MMP13, and that of its inhibitor TIMP1, was significantly increased in *Mdr2^−/−^* mice (Fig. 4B and C). MTA reduced MMP13 expression, and although not statistically significant there was a tendency towards decreased TIMP1 mRNA levels (Fig. 4B and C).

### MTA reduces hepatic levels of inflammatory and pro-fibrogenic mediators in *Mdr2^−/−^* mice

**Figure 5:**
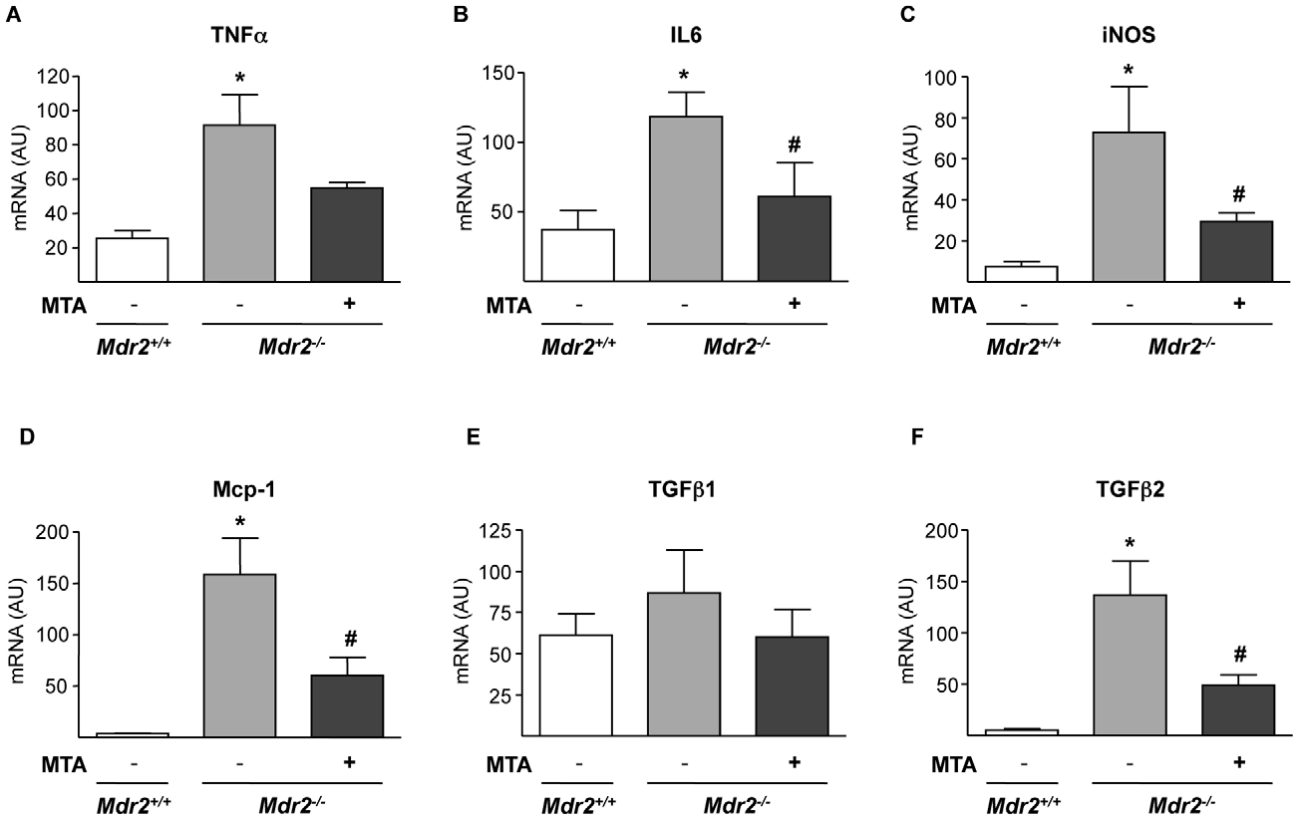
Effect of MTA on the expression of inflammatory cytokines and iNOS in *Mdr2^−/−^* mouse liver. mRNA levels of TNFα (A), IL6 (B), iNOS (C), Mcp-1 (D), TGFβ1 (E) and TGFβ2 (F) were measured in the liver of *Mdr2^+/+^*, control *Mdr2^−/−^*, and MTA-treated *Mdr2^−/−^* mice. *P<0.05 *vs Mdr2^+/+^* mice, #*P*<0.05 *vs* untreated *Mdr2^−/−^* mice.

**Figure 6:**
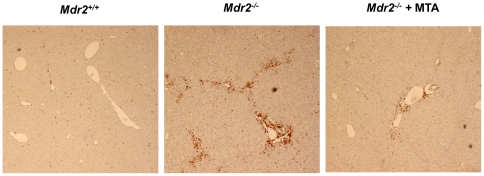
MTA reduces the recruitment of leukocytes to the portal fields of *Mdr2^−/−^* mice. Representative staining for the pan-leukocyte marker CD45 showing increased accumulation of CD45 positive cells in the portal field of *Mdr2^−/−^* mice compared to WT animals. Treatment with MTA reduced CD45 positive leukocyte accumulation. Representative images are shown.

Although the expression of inflammatory cytokines in the liver of *Mdr2^−/−^* mice is maximal between 2–8 weeks [10], [11], we still detected elevated TNFα and IL6 mRNA levels (Fig. 5A and B). MTA tended to reduce TNFα mRNA, and significantly lowered IL6 expression (Fig. 5A and B). Consistently, the expression of iNOS gene, a transcriptional target of pro-inflammatory cytokines, was decreased by MTA (Fig. 5C). Importantly, upon MTA administration we observed reduced Mcp-1 expression (Fig. 5D), a pro-fibrogenic chemokine up-regulated in *Mdr2^−/−^* mice [12]. TGFβ1 and TGFβ2 expression is elevated in the liver of these animals, particularly during the first 8 weeks of life [10]–[12]. Accordingly we found that by 11 weeks TGFβ1 expression in *Mdr2^−/−^* mice was similar to wild types, while TGFβ2 levels were still high, and were attenuated by MTA (Fig. 5E and F). These findings were accompanied by a significant decrease in inflammatory infiltrate, as assessed by staining for the leukocyte marker CD45 (Fig. 6).

**Figure 7:**
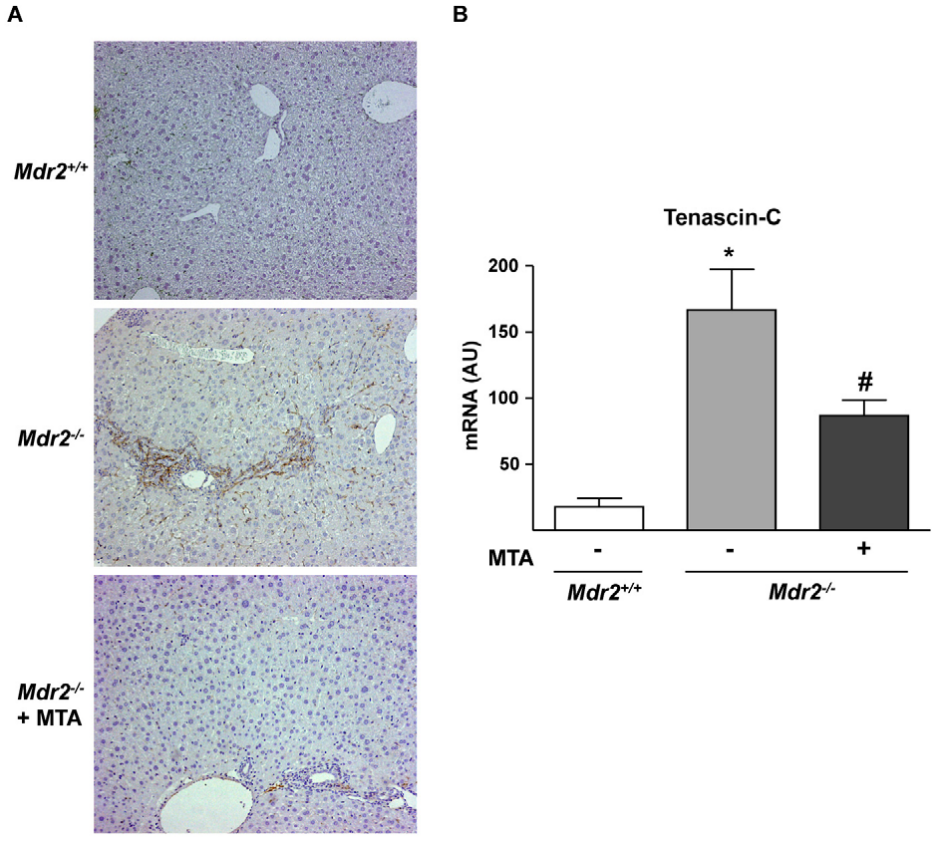
Expression of the fibrosis-associated protein tenascin-C, up-regulated in *Mdr2^−/−^* mice, is attenuated by MTA administration. Immunohistochemistry of tenascin-C in representative liver sections shows increased staining around portal areas in *Mdr2^−/−^* mice and its downregulation by MTA (A). Tenascin-C mRNA levels in the liver of *Mdr2^+/+^*, control *Mdr2^−/−^*, and MTA-treated *Mdr2^−/−^* mice (B). *P<0.05 *vs Mdr2^+/+^* mice, #*P*<0.05 *vs* untreated *Mdr2^−/−^* mice.

Tenascin-C is a TGFβ-inducible extracellular protein expressed in liver fibrogenic cells that participates in inflammation and fibrogenesis [24], [26]. As described in other models of chronic liver injury [24], we found increased tenascin-C staining around portal areas in *Mdr2^−/−^* mice (Fig. 7A). MTA treatment reduced tenascin-C immunostaining and mRNA levels (Fig. 7A and B). However, the expression of αvβ6 integrin, which is induced in *Mdr2^−/−^* mice biliary epithelia and binds tenascin-C [13], was unaffected by MTA (not shown).

### Effect of MTA on signalling pathways involved in fibrosis and hepatocellular proliferation in the *Mdr2^−/−^* mouse liver

**Figure 8:**
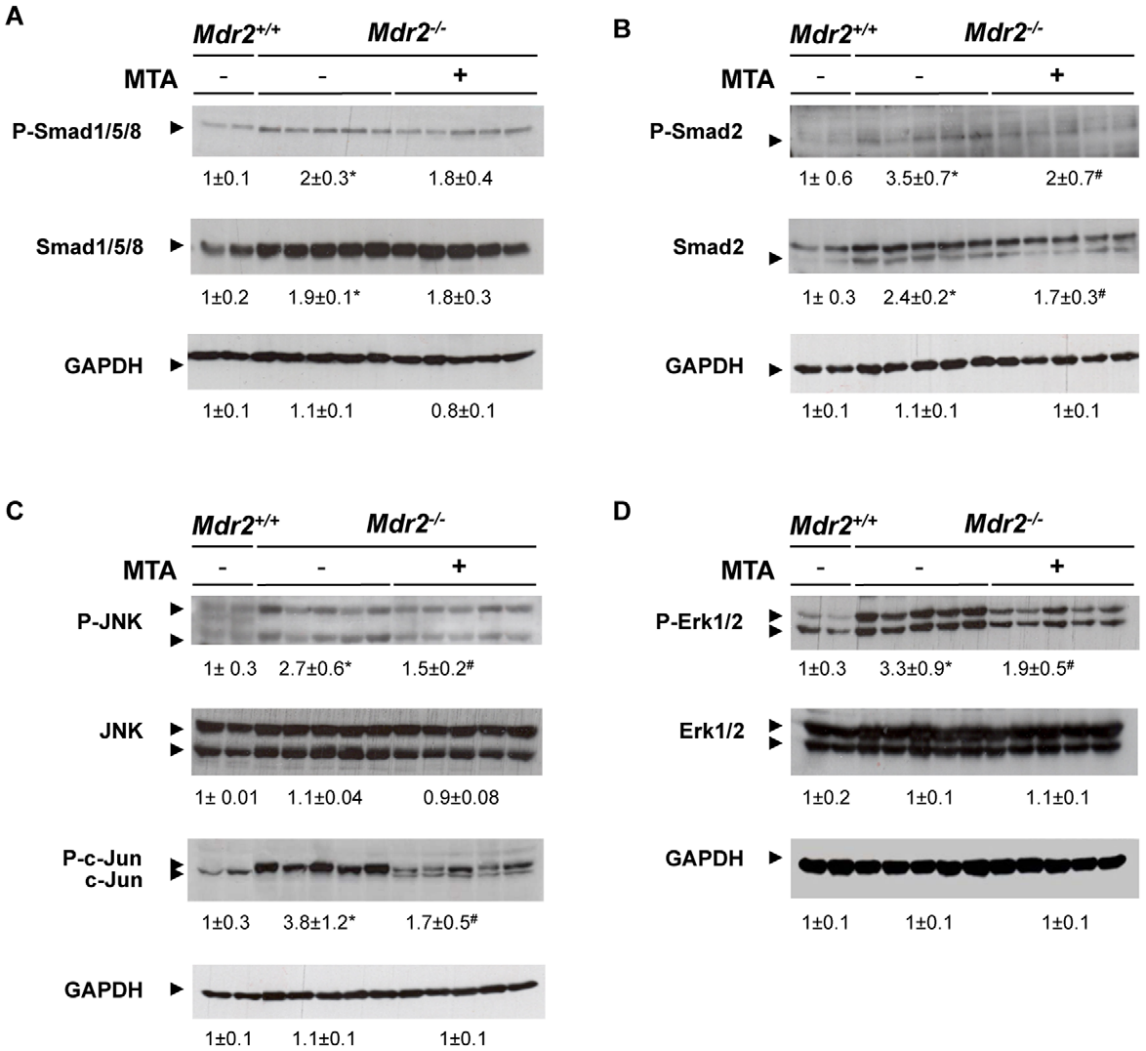
MTA modulates intracellular signalling pathways activated in *Mdr2^−/−^* mouse liver. Phosphorylation levels of Smad1/5/8 (A), Smad2 (B), JNK and c-Jun (C) and Erk1/2 (D) were analyzed by western blotting in liver extracts from *Mdr2^+/+^*, control *Mdr2^−/−^*, and MTA-treated *Mdr2^−/−^* mice. *P<0.05 *vs Mdr2^+/+^* mice, #*P*<0.05 *vs* untreated *Mdr2^−/−^* mice.

To further explore the mechanism of action of MTA we examined the activation of signalling pathways associated with fibrogenesis and disease progression, including the Smad and mitogen-activated protein kinase (MAPK) pathways [3], [6], [9], [27]–[30]. We found that total and phosphorylated Smad1/5/8 and Smad2 levels were increased in *Mdr2^−/−^* mouse liver extracts, and that MTA treatment reduced Smad2 and p-Smad2 contents (Fig. 8A and B). We also observed increased MAPK pathway activity in *Mdr2^−/−^* mice, as evidenced by elevated levels of p-Erk1/2, p-JNK and p-c-Jun. These pathways were also inhibited by MTA (Fig. 8C and D). No changes were observed in MTA-treated *Mdr2^+/+^* mice (not shown).

**Figure 9:**
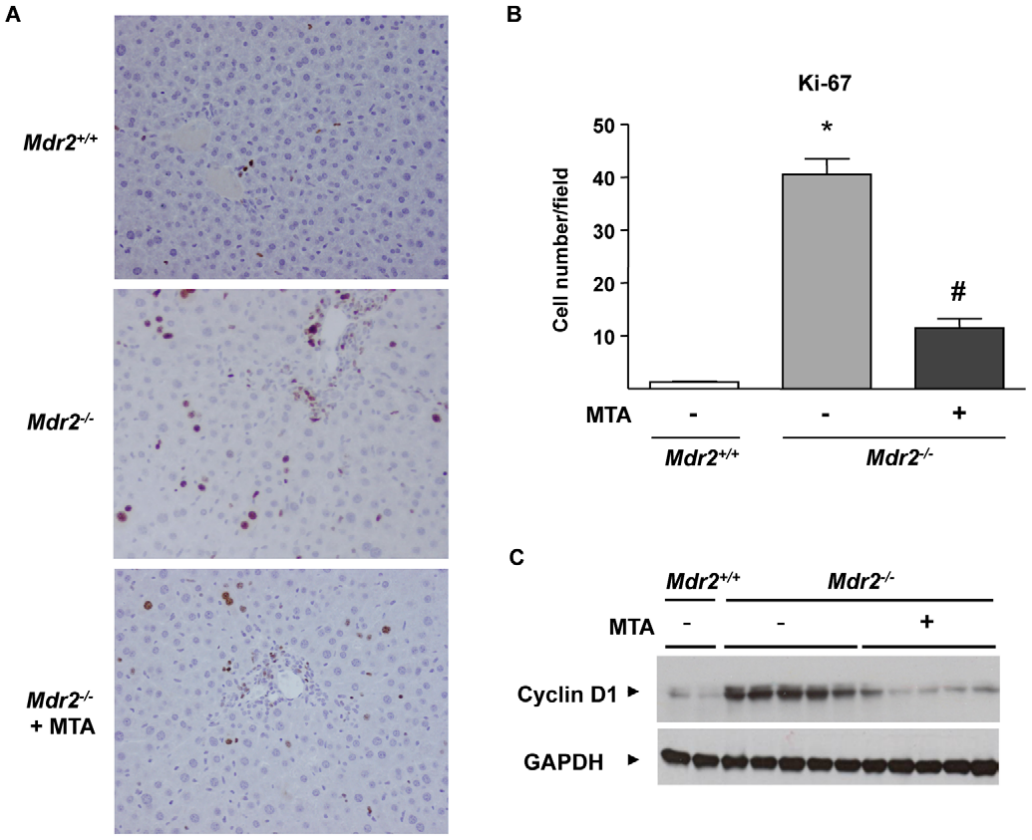
Increased hepatocellular proliferation in *Mdr2^−/−^* mice is attenuated by MTA administration. Immunohistochemical staining of Ki-67 in liver sections from *Mdr2^+/+^*, control *Mdr2^−/−^*, and MTA-treated *Mdr2^−/−^* mice (*Mdr2^−/−^*+MTA)(A). Ki-67 positive hepatocytes were counted in 30 high-power fields per mouse, n = 5 per group (B). *P<0.01 *vs Mdr2^+/+^* mice, #*P*<0.05 *vs* untreated *Mdr2^−/−^* mice. Western blot analysis of cyclin-D1 protein in liver extracts from *Mdr2^+/+^* mice, untreated *Mdr2^−^* ^/*−*^ and MTA-treated *Mdr2^−/−^* mice (C).

In agreement with the hyperactivation of pro-mitogenic signalling pathways, and confirming previous reports [6], [30], we found enhanced hepatocellular proliferation in *Mdr2^−/−^* mice, as evidenced by increased hepatocyte staining with the proliferation marker Ki-67 (Fig. 9A and B). MTA reduced hepatocellular proliferation (Fig. 9A and B) and cyclin D1 protein levels (Fig. 9C), markedly elevated in the liver parenchymal cells of these animals [14].

### MTA effects on liver SAMe and GSH levels in *Mdr2^−/−^* mice

*In vivo* MTA is produced from SAMe during polyamine metabolism, and subsequently MTA can be metabolically recycled back to methionine [15], [31]. Methionine metabolism in turn is necessary for the production of SAMe and GSH, and it is impaired in liver injury [31]. The expression of key genes in methionine metabolism is down-regulated in *Mdr2^−/−^* mice [32]. In view of this we measured MTA effects on liver SAMe levels. We observed that SAMe was not reduced in *Mdr2^−/−^* mice, and was unaffected by MTA treatment (82.9±10.6 in *Mdr2^+/+^*; 110.6±9.7 in *Mdr2^−/−^* and 103.2±12.3 in MTA-treated *Mdr2^−/−^*, values are nmol/g tissue). GSH levels were not altered either in *Mdr2^−/−^* mice compared to *Mdr2^+/+^*, and were not affected by MTA (6.49±0.57 in *Mdr2^+/+^*; 5.92±0.68 in *Mdr2^−/−^* and 5.61±0.51 in MTA-treated *Mdr2^−/−^*, values are µmol/g tissue).

### Effects of MTA on inflammatory cells

**Figure 10:**
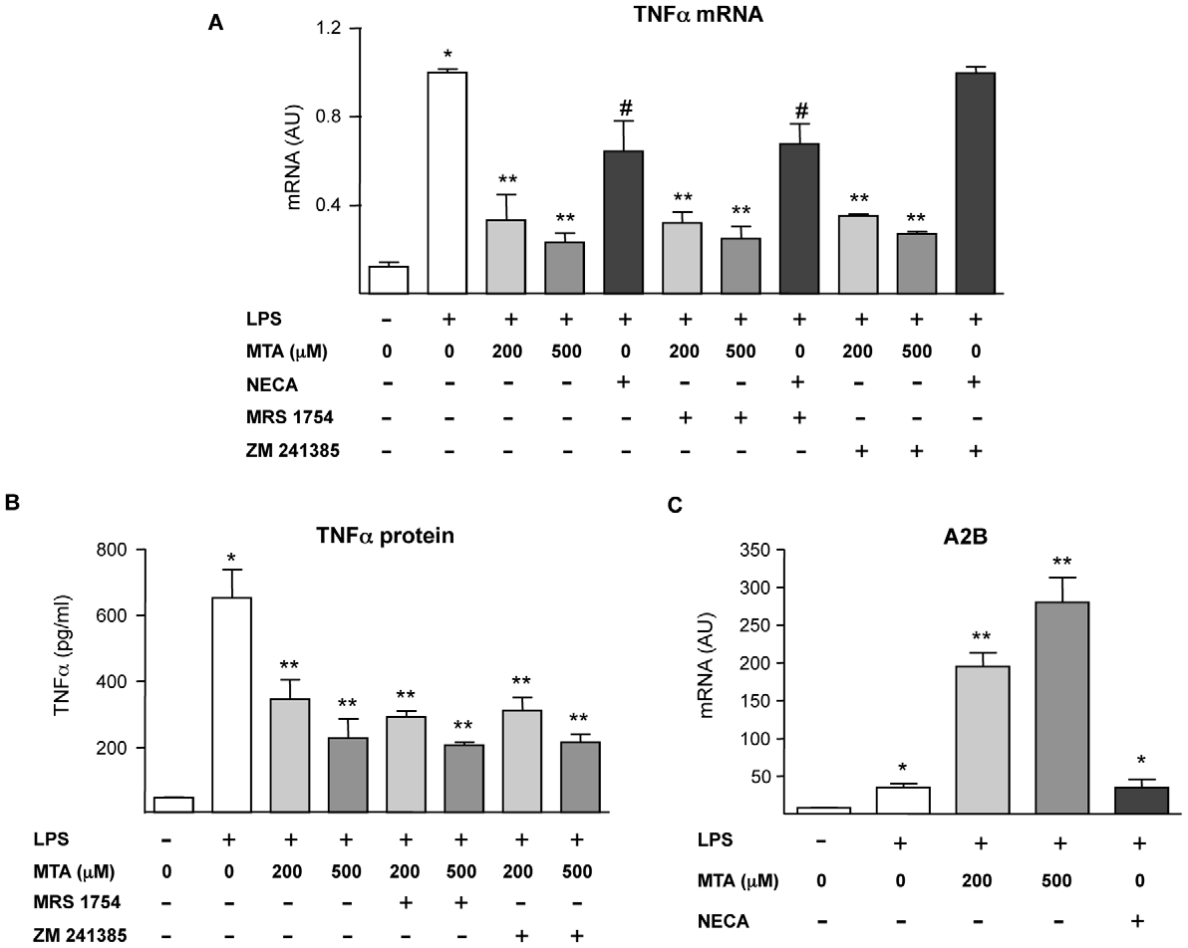
Effect of MTA on the activation of inflammatory cells: interaction with adenosine receptors. As indicated in the figure serum-starved murine monocyte/macrophages RAW 264.7 cells were pre-treated for 30 min with the A2A receptor antagonist ZM 241385 (10 µM) or the A2B receptor antagonist MRS 1754 (1 µM), then MTA or the adenosine receptor agonist NECA (20 µM) where added to the cultures for another 30 min. Subsequently and where indicated cells were treated with LPS (50 ng/ml) for up to 5 h. TNFα mRNA levels in cell lysates (A) and TNFα protein contents in conditioned media (B) were measured. *P<0.01 *vs* control, **P<0.01 *vs* LPS, #*P*<0.05 *vs* LPS. The expression of the adenosine A2B receptor was measured in RAW 264.7 cells pre-treated with MTA or the adenosine agonist NECA (20 µM) for 30 min and then where indicated with LPS (50 ng/ml) for up to 5 h (C). *P<0.01 *vs* control, **P<0.01 *vs* LPS.

Our current *in vivo* observations indicate that MTA administration significantly attenuated the inflammatory response found in the liver of *Mdr2−/−* mice. MTA is structurally very close to adenosine, a potent endogenous immune regulatory molecule produced mainly by cells of the immune system during tissue injury and inflammation [33]. Adenosine mediates its cellular effects through the interaction with cell surface receptors such as A2A and A2B, which are expressed in monocytes/macrophages and play a major role in the anti-inflammatory and immunomodulatory actions of this nucleoside [33], [34]. In view of this and considering the ability of MTA to interact with adenosine receptors [35], we decided to explore the potential implication of A2A and A2B receptors in MTA anti-inflammatory effects. To directly address this issue we took advantage of the murine monocyte/macrophage cell line RAW 264.7, which has been widely used in mechanistic studies on the anti-inflammatory effects of adenosine upon bacterial lipopolysaccharide (LPS) challenge [36], [37]. First we evaluated the expression and release of TNFα in LPS-stimulated RAW 264.7 cells in the presence of MTA. As shown in Fig. 10A, and in agreement with our previous findings [16], we found a potent inhibitory effect of MTA on TNFα expression. We also observed that the inhibitory effect of MTA was preserved when RAW 264.7 cells were incubated in the presence of the A2A or A2B specific antagonists ZM 241385 and MRS 1754 respectively (Fig. 10A and B). On the other hand, and in accordance with the literature [38], [39], the inhibition of LPS-elicited TNFα production by the stable adenosine analogue NECA was attenuated by the A2A antagonist ZM 241385 (Fig. 10A). These observations suggested that the effect of MTA on the activation of inflammatory cells was not dependent on its potential direct interaction with adenosine receptors. However, although apparently MTA was not acting as an A2B receptor agonist we found that this molecule dramatically synergized with LPS in the up-regulation of A2B mRNA levels (Fig. 10C). Increased A2B expression has been linked to the activation of anti-inflammatory responses [37], [39], [40]. Interestingly this effect of MTA was not reproduced by the adenosine analogue NECA (Fig. 10C).

### Effect of MTA on cultured liver myofibroblasts

**Figure 11:**
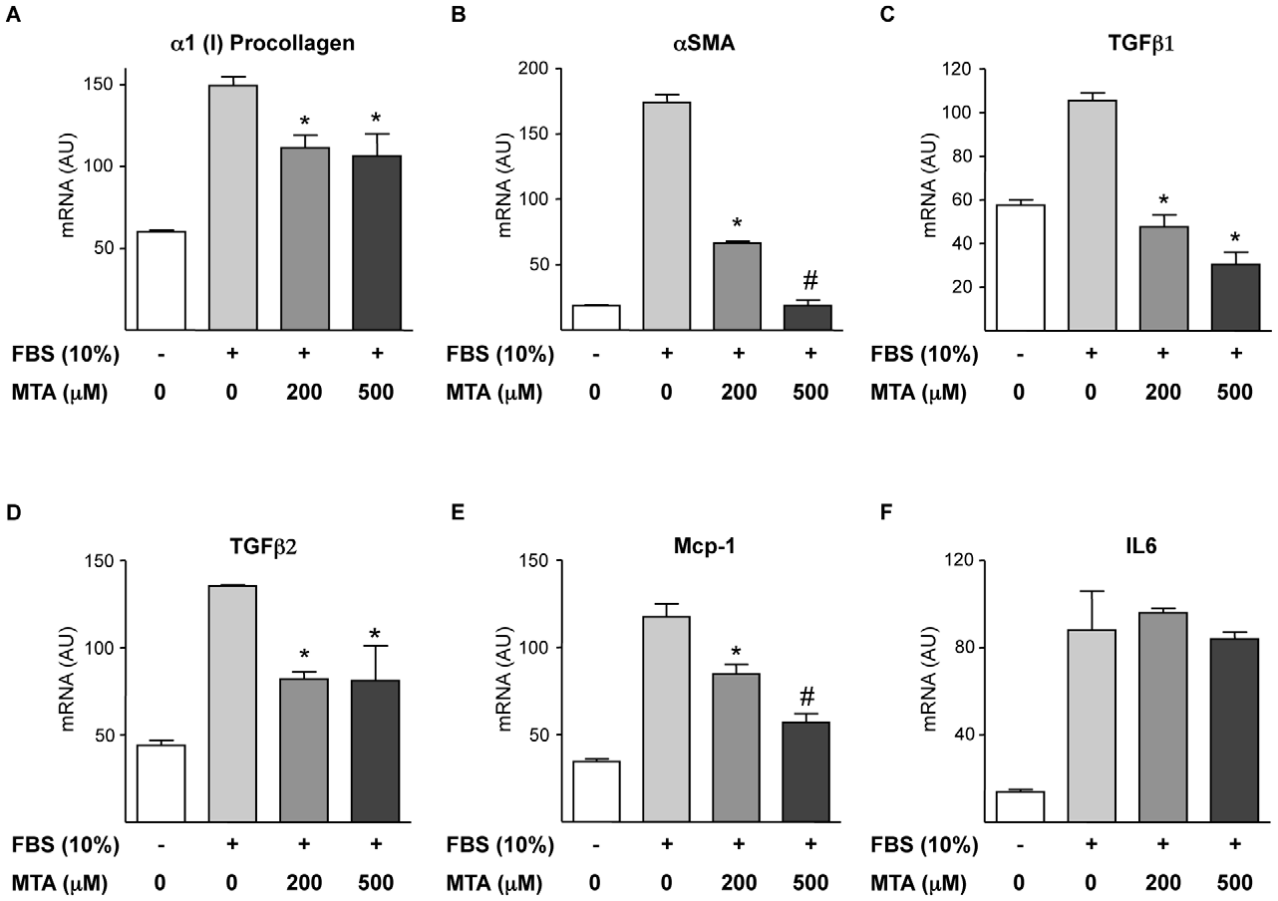
Effects of MTA on liver myofibroblast activation and cytokine expression. Primary myofibroblasts were serum starved (12 h) and then treated (12 h) with 10% FCS and MTA as indicated. mRNA levels of α1(I)procollagen (A), αSMA (B), TGFβ1 (C), TGFβ2 (D), Mcp-1 (E) and IL6 (F) were measured at the end of treatments. *P<0.05 *vs* FCS, #*P*<0.05 *vs* FCS+MTA 200 µM.

Next we tested whether MTA had a direct effect on liver myofibroblasts, which participate in biliary fibrosis and represent a relevant source of extracellular matrix in *Mdr2^−^* ^/*−*^ mice [9], [30], [41]. Given that isolated myofibroblasts display high proliferation and activity upon *in vitro* culture, we pre-incubated the cells in serum free medium for 12 h to attenuate activation and proliferation, and subsequently cultures were stimulated with 10% FCS to promote myofibroblast activation in the presence or absence of MTA. We observed that the expression of the fibrogenic markers α1(I)procollagen and αSMA elicited by FCS treatment was inhibited by MTA (Fig. 11A and B). Similar observations were made when myofibroblasts were challenged with TGFβ (not shown). Additionally, TGFβ1 and TGFβ2 expression triggered by FCS was also attenuated by MTA (Fig. 11C and D). In agreement with previous reports [30] we found that FCS treatment increased Mcp-1 expression, and this response was also reduced by MTA (Fig. 11E), while IL6 expression was unaltered (Fig. 11F).

**Figure 12:**
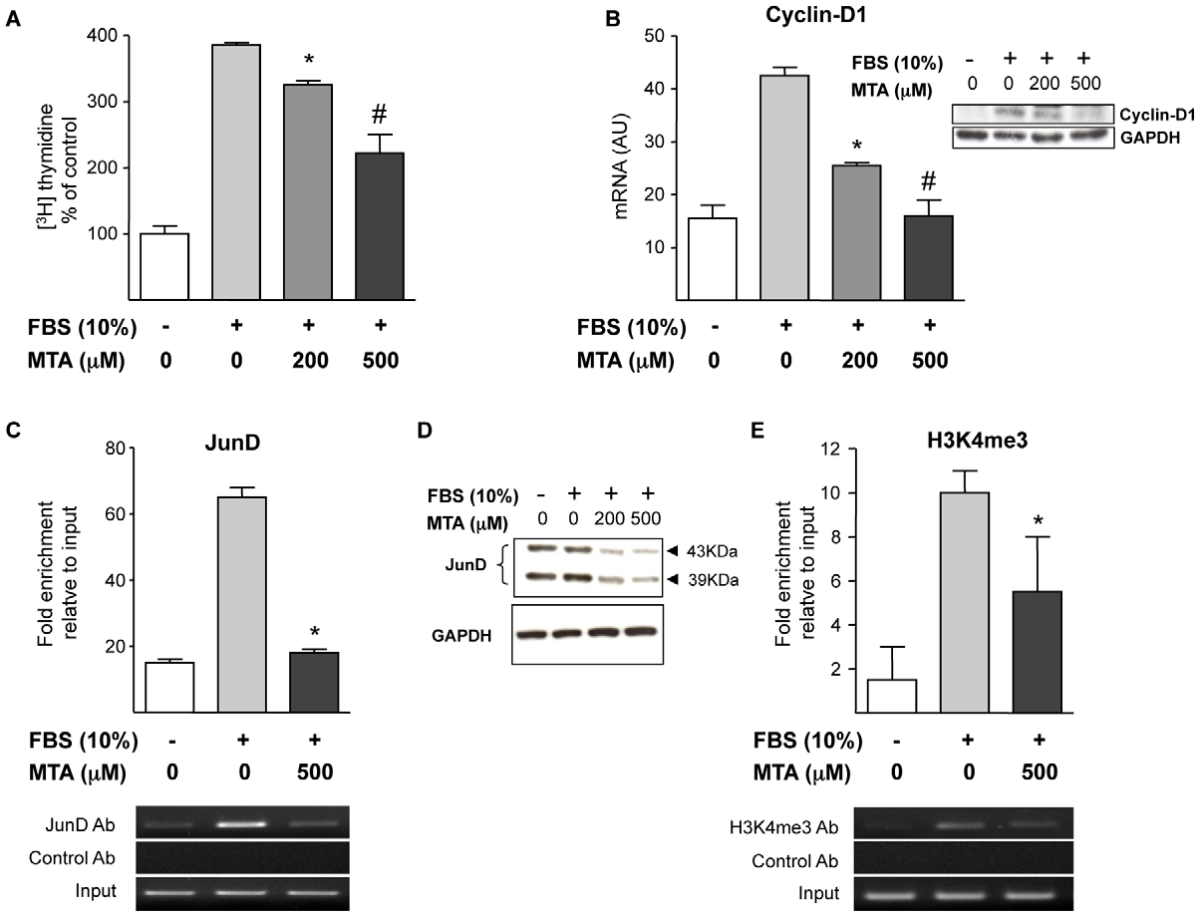
MTA inhibits DNA synthesis in myofibroblasts. MTA Effects on cyclin D1 and JunD expression. Effect of MTA on FCS-induced [^3^H]thymidine incorporation in myofibroblasts treated as indicated (24 h) (A). Cyclin D1 mRNA expression in myofibroblasts treated (12 h) with 10% FCS and MTA as indicated. Inset shows a Western blot analysis of cyclin D1 protein (B). ChIP assay measuring the binding of JunD to the −905 to −725 cyclin D1 promoter region encompassing a key AP-1 binding site in myofibroblasts treated as indicated (12 h). Immunoprecipitated genomic DNA corresponding to the −905 to −725 cyclin D1 promoter region was quantified by real time PCR as indicated in Methods section. A representative gel picture of amplified DNA fragments is shown (C). Western blot analysis of JunD protein levels in myofibroblasts treated as indicated for 6 h (D). ChIP assay measuring the binding of tri-methylated-H3K4 (H3K4me3) to the same cyclin-D1 promoter region in myofibroblasts treated as indicated (12 h). A representative gel picture of amplified DNA fragments is shown (E). *P<0.05 *vs* FCS, #*P*<0.05 *vs* FCS+MTA 200 µM.

We also examined whether MTA could affect myofibroblast proliferation. Figure 12A shows that MTA reduced FCS-triggered DNA synthesis, without causing any toxicity (determined by lactate dehydrogenase release, not shown). Additionally we observed that MTA did not induce apoptosis in cultured myofibroblasts (data not shown). Our [^3^H]thymidine incorporation experiments suggested that MTA inhibited cell cycle progression through the S phase (DNA synthesis), therefore we examined the expression of cyclin D1, a key cyclin in the transition from G_0_/G_1_ to S phase. We found that cyclin D1 expression was reduced by MTA treatment both at the mRNA and protein levels (Figure 12B). The expression of cyclin D1 can be regulated by AP-1 transcription factors, and among the components of the AP-1 complex JunD has been identified as a critical transcription factor in liver fibrogenesis [27], [42]. Therefore we examined the binding of JunD to a region of the cyclin D1 promoter that contains a key AP-1 binding site [25] by ChIP analysis. As depicted in Figure 12C, MTA treatment almost completely blunted JunD association with cyclin D1 promoter in this regulatory region. In view of this response we examined the expression of JunD in cultured myofibroblasts treated with MTA, and observed that MTA significantly reduced JunD protein levels (Fig. 12D). These data suggested that the reduction of cyclin D1 expression by MTA could be mediated in part through the downregulation of JunD levels. Nevertheless, in spite of this effect of MTA on JunD cellular contents this compound almost totally inhibited cyclin D1 expression, and our ChIP experiment showed a near complete inhibition of JunD binding to the cyclin D1 promoter. This suggested that MTA could also affect the interaction of the remaining JunD protein with this cyclin D1 regulatory region. MTA is a well known inhibitor of methylation reactions [15], and it has been shown to inhibit H3K4 trimethylation, a hallmark of open chromatin conformation and transcriptional activation [43], in different cell types [18], [44]. In view of this we examined the levels of tri-methylated-H3K4 in chromatin associated with this region of the cyclin D1 promoter. We detected increased levels of tri-methylated-H3K4 upon FCS stimulation, and observed that this modification was lowered by MTA treatment (Fig. 12E).

**Figure 13:**
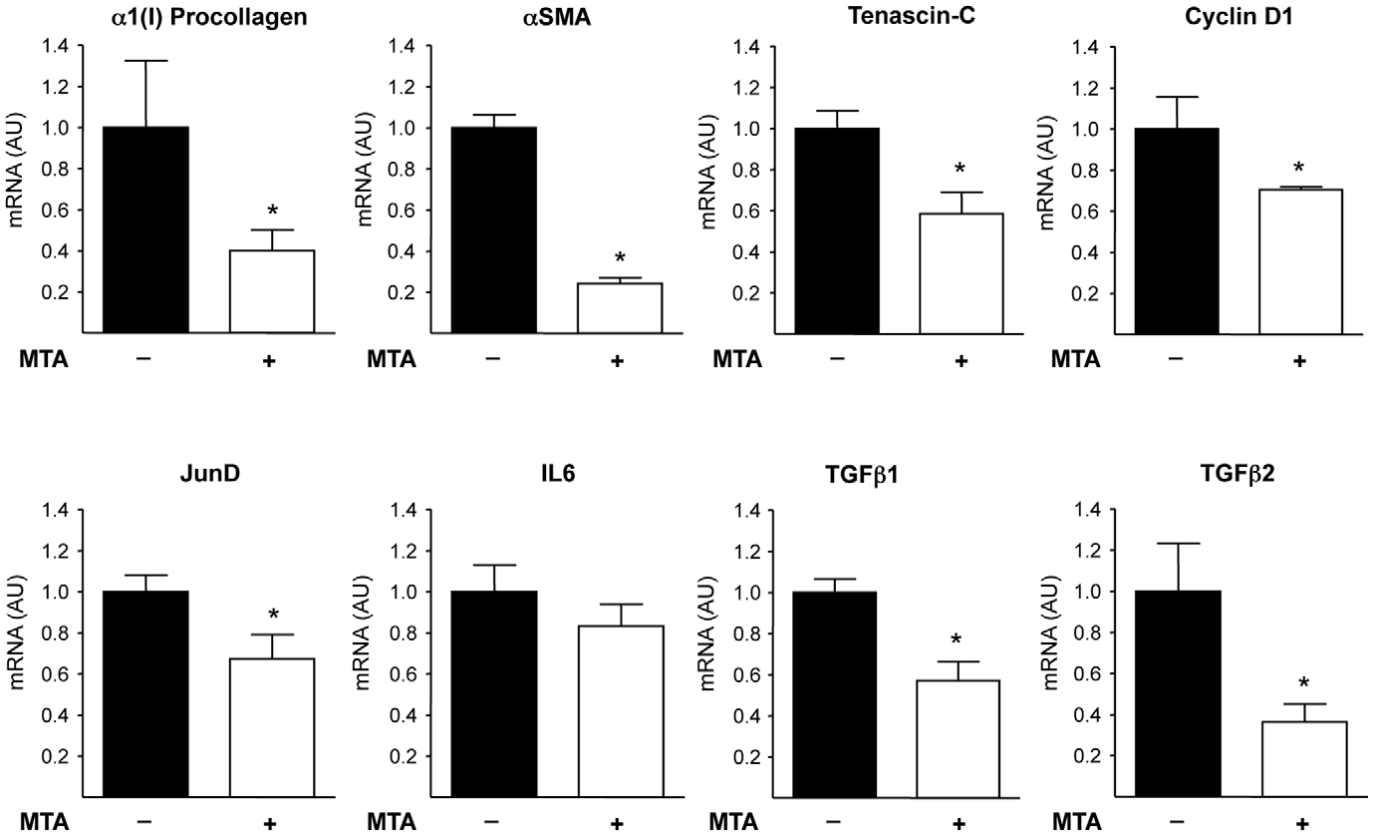
Expression of fibrogenic activation and cellular proliferation-related genes in myofibroblasts isolated from the liver of control and MTA-treated *Mdr2−/−* mice. *Mdr2−/−* mice were treated for three weeks with MTA or vehicle as described in the Methods section. At the end of the treatments hepatic myofibroblasts were isolated and the mRNA levels of the indicated genes were measured. Data are means ±SEM of three independent cell preparations per condition. *P<0.05 *vs* vehicle-treated *Mdr2−/−* mice.

The relevance of these *in vitro* effects of MTA on the expression of fibrogenic and cell proliferation-related genes was supported by the observation of reduced α1(I)procollagen, αSMA, tenascin-C, TGFβ1, TGFβ2, JunD and cyclin D1 expression in myofibroblasts isolated from *Mdr2−/−* mice treated for 3 weeks with MTA when compared to cells isolated from untreated mice (Fig.13).

**Figure 14:**
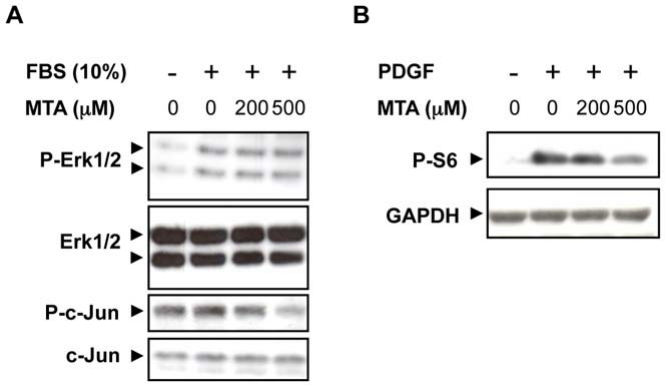
Effect of MTA on liver myofibroblast intracellular signalling pathways. Liver myofibroblasts were pre-treated with MTA for 30 min in the absence of serum and then stimulated with 10% FCS for 30 min or PDGF (20 ng/ml) for 60 min. Phosphorylation levels of Erk1/2 and c-Jun upon FCS stimulation (A), and S6 ribosomal protein after PDGF treatment (B) were analyzed by western blotting. Representative blots are shown.

Finally, to gain additional insight into the mechanisms of the antifibrogenic effects of MTA we explored the direct effects of this molecule on the activity of intracellular signalling pathways related to the proliferation and fibrogenic activation of myofibroblasts [27]–[30]. As depicted in Fig. 14A, while MTA treatment was not able to directly modify the phosphorylation of Erk1/2 elicited by FCS, it reduced the levels of phospho-c-Jun, a target of the pro-fibrogenic JNK signalling pathway in liver extracellular matrix producing cells [29]. Interestingly, MTA significantly attenuated the phosphorylation of the mTOR and p70^S6^ kinase downstream target S6 ribosomal protein triggered by the pro-fibrogenic growth factor PDGF (Fig. 14B). p70^S6^ kinase activity is required for G_1_ cell cycle progression, and it has been also identified as a signal transducer for liver fibrogenic cell growth [45].

## Discussion

We have shown that oral administration of the nucleoside MTA significantly ameliorates liver injury in *Mdr2^−/−^* mice. These mice represent a clinically relevant model of spontaneous chronic liver disease, spanning from peribiliary inflammation and fibrosis to HCC development [9]–[14]. MTA was well tolerated and a significant improvement in serum markers of hepatic injury and function was observed. This was accompanied by reduced hepatic fibrosis and fibrogenic cell abundance. Accordingly, α1(I)procollagen mRNA levels were lowered, and TIMP1 expression tended to decrease. The fact that MTA also reduced the expression of MMP13, the interstitial collagenase in rodents, suggests that MTA down-regulates overall tissue remodelling activity, inhibiting fibrosis progression rather than stimulating fibrosis resolution [3].

Previous studies have shown that MTA exerts remarkable biological effects, its pharmacologic administration modulates different pathways and cellular responses, including gene expression, cell cycle progression and inflammation [15]–[20]. Therefore, the mechanisms behind MTA therapeutic action on a complex model such as *Mdr2^−/−^* mice are likely multifaceted. Our current findings agree with those reported by Simile et al., who showed that parenteral MTA administration to rats undergoing chronic CCl_4_-induced liver injury reduced fibrosis [19]. This effect was mechanistically related to MTA antioxidant properties, that counteracted the oxidative stress and subsequent injury generated by CCl_4_ metabolism [19]. However, oxidative stress is not a prominent feature in *Mdr2^−/−^* mice [14]. Accordingly, we found that GSH levels were unaltered in *Mdr2^−/−^* mouse liver, and were not affected by MTA, suggesting that MTA effects were not attributable to its antioxidant potential. *In vivo* MTA is metabolized to methionine in the so called methionine salvage pathway, and in the liver methionine is efficiently metabolized into SAMe [15]. SAMe is a key methyl donor with significant hepatoprotective properties, including antifibrotic activity in experimental cholestasis [31], [46]. In view of the metabolic links between MTA and SAMe, we measured SAMe levels in control and MTA-treated *Mdr2^−/−^* mouse liver but did not observe major differences, indicating that MTA effects were not likely mediated through its potential contribution to the SAMe pool. Moreover, this may suggest that the antifibrotic effects of SAMe in experimental cholestasis [46] could be mediated in part by the metabolic or non-enzymatic conversion of SAMe into MTA [15].

Interestingly, MTA reduced the expression of the extracellular matrix protein tenascin-C in the liver of *Mdr2^−/−^* animals. This may be directly related to MTA antifibrotic effects, since tenascin-C is required for the development of experimental liver fibrosis [24]. Furthermore, tenascin-C is up-regulated in chronic hepatitis C, and its expression in peritumoral activated stellate cells in HCC associates with poor clinical prognosis [26], [38], [47]. In view of this, tenascin-C may be a previously unrecognized player in fibrosis and HCC development in *Mdr2^−/−^* mice, and its attenuation by MTA a key event in the anti-fibrotic and anti-neoplastic properties of this molecule [20]. Moreover, tenascin-C binds and activates αvβ6 integrin, a recently identified pharmacological target in biliary fibrosis [13].

As occurs in human chronic liver disease, progression of liver injury in *Mdr2^−/−^* mice is associated with persistent inflammation. The observation that anti-inflammatory compounds like ibuprofen or curcumin ameliorate injury and fibrosis in *Mdr2^−/−^* mice [6], [30], and that TNFα blockade prevents hepatocarcinogenesis in these animals [6], underscore the role played by inflammation in this model. We and others have previously shown that MTA has a significant immunomodulatory potential. MTA inhibits NF-κB mediated signalling, reduces inflammatory cytokine production by macrophages and lymphocytes, and attenuates the cellular effects of these cytokines [16]–[18]. We now observed that in *Mdr2^−/−^* mice MTA reduced the expression of IL6, Mcp-1, the NO-generating enzyme iNOS, and the profibrogenic factor TGFβ2. Accordingly MTA attenuated the activation of Erk1/2, JNK and Smad2, intracellular pathways triggered by these cytokines and which are directly implicated in fibrogenesis [3], [5], [27]–[30]. Therefore, interference with inflammatory cytokine and NO generation likely contributes to MTA hepatoprotective and anti-fibrogenic effects in this model [10], [48].

From a mechanistic perspective, and in view of the remarkable anti-inflammatory effects of MTA, we cannot overlook the fact that this molecule is structurally very close to adenosine, a purine nucleoside released form cells with notorious anti-inflammatory properties [15], [33]. Adenosine interacts with different G-protein-coupled cell surface receptors including the A1, A2A, A2B and A3 receptors. Until recently adenosine anti-inflammatory effects were mainly attributed to its interaction with the high affinity A2A receptor subtype [49], however an important role for the low affinity A2B receptor in the anti-inflammatory response to adenosine is currently emerging [34], [38], [40]. Using specific pharmacological antagonists of the A2A and A2B receptors we evaluated their potential implication in the immunomodulatory effects of MTA on mouse RAW 264.7 monocytes/macrophages challenged with bacterial LPS, a prototypic model of the inflammatory response. We found that the inhibition of LPS-elicited TNFα production by MTA was not impaired in the presence of these drugs, suggesting that MTA effects were not mediated through these receptors. Nevertheless, the involvement of the adenosine signalling system in the biological effects of MTA cannot be completely disregarded. Interestingly we observed that MTA treatment resulted in a remarkable potentiation of the expression of A2B receptors triggered by LPS, and this effect was not shared by the adenosine receptor agonist NECA. As previously mentioned A2B receptors display low affinity for adenosine, but their expression is up-regulated during tissue injury and inflammation, conditions in which interstitial adenosine concentrations raise. The expression of A2B receptors seems to be very important for the attenuation of the systemic inflammatory response, as illustrated by the fact that A2B knockout mice show enhanced cytokine production and overall mortality during sepsis [39], [50]. Therefore the stimulatory effect of MTA on A2B receptor expression could also contribute to dampen inflammation in the chronically injured *Mdr2−/−* mouse liver.

In addition to these responses, a direct cytoprotective effect of MTA on hepatocytes may also be involved, as we previously observed that MTA can prevent hepatocyte death *in vitro* [51], [52]. Mechanistically, the attenuation of JNK activity found in MTA-treated *Mdr2^−/−^* mice could be related to the hepatoprotective effects of this molecule, given that inhibition of JNK has been demonstrated to prevent liver damage in different *in vitro* and *in vivo* experimental models of hepatocellular injury [53]–[55]. In this context the reduced hepatocyte proliferation found in MTA-treated mice may be related in part to the attenuation of liver damage, since liver regeneration occurs in response to injury.

We also observed that MTA has direct effects on liver fibrogenic cells. A previous study had reported that MTA could reduce collagen gene expression in primary hepatic stellate cells [56]. Here we confirmed this observation in liver myofibroblasts, and we also found that MTA down-regulated also the expression of αSMA, TGFβ1 and TGFβ2, and Mcp-1, all markers of activated fibrogenic cells [3], [6], [57]. However, MTA did not modify IL6 mRNA levels in myofibroblasts. This supports the specificity of MTA effects on gene expression, and indicates that the downregulation of IL6 observed *in vivo* could be attributed to the interaction of MTA with other cell types, likely inflammatory cells. The reduction of TGFβ isoforms and Mcp-1 expression by MTA found *in vivo* as well as in cultured myofibroblasts may be important effects regarding the mechanisms of action of this molecule. TGFβ1 and TGFβ2 are key cytokines in the development of liver fibrosis that can be expressed in a variety of cells including activated stellate cells, myofibroblats, macrophages and activated cholangiocytes [58], [59]. The inhibition of TGFβ1 and TGFβ2 expression and activation has been demonstrated to attenuate liver fibrosis, including biliary fibrosis [59]. On the other hand, Mcp-1 participates in the recruitment of inflammatory cells during liver injury, and has been cogently demonstrated to contribute to hepatic parenchymal damage [60]. Moreover, besides expressing Mcp-1 liver fibrogenic cells can be activated and induced to proliferate by this chemokine [61], [62]. Together these findings suggest that attenuation of TGFβ1/2 and Mcp-1 expression could have important mechanistic roles in the therapeutic action of MTA.

Increased proliferation and survival of ECM-producing cells are central events in the development of liver fibrosis [5], [58]. MTA has been shown to induce apoptosis of different cell types, including HCC cells [51]. However, we did not observe any pro-apoptotic or cytotoxic effect of MTA in liver myofibroblasts. What we found was a significant inhibition by MTA of FCS-stimulated DNA synthesis. In line with this response we observed that MTA reduced the expression of cyclin D1, a key G_1_ cyclin associated with liver fibrogenic cell proliferation [63]. AP-1 transcription factors can stimulate the expression of genes involved in the activation and proliferation of liver fibrogenic cells, and among them JunD is known to play an important role in liver fibrogenesis [27], [42]. To gain further insight into the mechanism of action of MTA, we examined the binding of JunD to cyclin D1 promoter and found that it was markedly reduced in myofibroblasts treated with this compound. This can be due in part to the observed downregulation of JunD levels in MTA-treated cells, and therefore reduction of JunD expression may be a relevant mechanism in the antifibrogenic action of MTA.

In order to better understand the mechanisms behind the antiproliferative effects of MTA, we examined the activity of three signalling pathways that are involved in fibrogenic cell proliferation. We found that while MTA did not affect FCS-triggered Erk1/2 phosphorylation, it reduced phopho-c-Jun levels and significantly attenuated PDGF elicited ribosomal S6 protein phosphorylation. Both, the JNK and the mTOR and p70^S6^ kinase pathways have been demonstrated as key regulators of hepatic fibrogenic cell proliferation [29], [45], and therefore their inhibition by MTA could be relevant in the mechanism of action of this compound. The selective interaction of MTA with protein kinase-mediated intracellular signalling pathways as described here is likely to have profound effects on cellular behaviour and thus deserves further examination.

**Figure 15:**
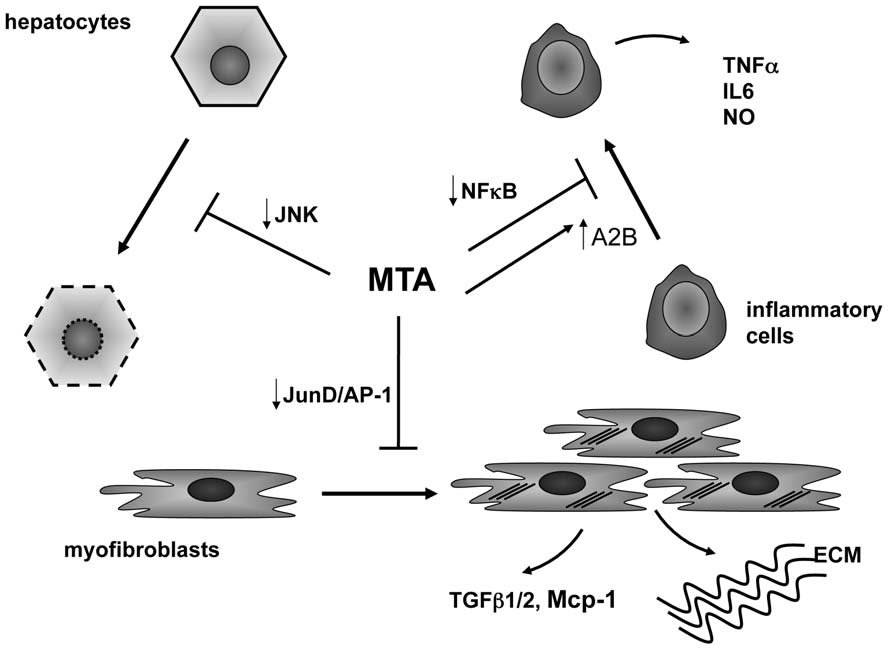
Potential mechanisms of action of MTA in *Mdr2^−/−^* mice. Our *in vivo* and *in vitro* observations indicate that MTA displays different actions that may underlie its beneficial effects on the course of liver injury and fibrosis in *Mdr2^−/−^* mice. MTA may exert a direct cytoprotective effect on hepatocytes by reducing JNK activity, preventing hepatocellular death and further inflammation. MTA also inhibits the production of cytokines by inflammatory cells, likely through interference with NFκB activity as described before [16]–[18]. In addition, enhanced expression of adenosine A2B receptors may contribute to the anti-inflammatory pharmacological profile of MTA. This compound can also exert direct effects on extracellular matrix (ECM) producing cells (myofibroblasts), reducing their activation, proliferation and the production of pro-fibrogenic factors. Inhibition of JunD expression may be an important event in the antifibrogenic action of MTA.

We also noticed that MTA could attenuate the increase in H3K4 trimethylation elicited by FCS stimulation in the cyclin D1 promoter. H3K4 trimethylation is a marker of transcriptional active chromatin, and this modification contributes to gene activation [64]. MTA is an inhibitor of methylation reactions [15], including histone methylation [18], our observations suggest that interference with this epigenetic mechanism could account in part for the effects of MTA on cyclin D1 gene expression. Moreover, this finding makes it tempting to speculate that MTA could exert its antifibrogenic effects also through an overall action on the epigenetic programming. In this regard 3 deazaneplanocin, another methyltransferase inhibitor, has been recently reported to prevent the activation of liver fibrogenic cells, however its efficacy and tolerability in chronic *in vivo* models of liver fibrosis have not been tested yet [65]. Together these findings suggest that the antifibrogenic effects of MTA observed *in vivo* may be mediated in part through the direct inhibition of myofibroblast activation and proliferation. Nevertheless, a direct effect of MTA on bile duct epithelial cell damage and cholangiocyte activation, important events in the pathogenesis of liver injury in *Mdr2^−/−^* mice [30], cannot be discarded and merit future evaluation. Figure 15 summarizes the proposed mechanisms of action of MTA in *Mdr2^−/−^* mice mainly based on the observations reported in this study.

In summary, here we have demonstrated that oral MTA administration is an effective therapeutic strategy to attenuate disease progression in a relevant model of liver injury and fibrosis. In addition to our current study, MTA has been previously tested in *in vivo* experimental models never showing untoward effects [16], [17], [66], [67], and an ID_50_ of 2.9±0.4 g/kg was estimated when administered intramuscularly to rats [19]. In humans MTA administration is also well tolerated [68], [69]. These considerations, together with the efficacy described herein, suggest that MTA could be a good candidate to be clinically tested in the context of liver injury and fibrosis.
